# The use of reference gene selection programs to study the silvering transformation in a freshwater eel *Anguilla australis*: a cautionary tale

**DOI:** 10.1186/1471-2199-11-75

**Published:** 2010-09-22

**Authors:** Alvin N Setiawan, P Mark Lokman

**Affiliations:** 1 Department of Zoology, University of Otago, 340 Great King Street, P. O. Box 56, Dunedin 9054, New Zealand

## Abstract

**Background:**

Quantitative real-time PCR (qPCR) has been the method of choice for the quantification of mRNA. Due to the various artifactual factors that may affect the accuracy of qPCR, internal reference genes are most often used to normalize qPCR data. Recently, many studies have employed computer programs such as *GeNorm*, *BestKeeper *and *NormFinder *in selecting reference genes, but very few statistically validate the outcomes of these programs. Thus, in this study, we selected reference genes for qPCR of liver and ovary samples of yellow (juvenile), migratory (silver) and 11-KT treated juveniles of New Zealand shortfinned eels (*Anguilla australis*) using the three computer programs and validate the selected genes statistically using *REST 2009 *software and the Mann-Whitney test. We also tested for the repeatability of use for the best reference genes by applying them to a data set obtained in a similar experiment conducted the previous year.

**Results:**

Out of six candidate genes, the combination of *18 s *and *eef1 *was found to be the best statistically validated reference for liver, while in ovary it was *l36*. However, discrepancies in gene rankings were found between the different programs. Also, statistical validation procedures showed that several genes put forward as being the best by the programs were in fact, regulated, making them unsuitable as reference genes. Additionally, *eef1 *which was found to be a suitable - though not the top ranked - reference gene for liver tissues in one year, was regulated in another.

**Conclusions:**

Our study highlights the need for external validations of reference gene selections made by computer programs. Researchers need to be vigilant in validating and reporting the rationale for the use of reference gene in published studies.

## Background

Quantitative real-time PCR (qPCR) has by far been the most widely used method for the measurement of transcript abundance. The method is applicable across most tissues and targets of interest, sensitive, relatively inexpensive and quick compared to Northern blotting. Most importantly, when employed appropriately, it allows for accurate quantification over a wide dynamic range of template amounts.

However, the level of accuracy depends upon various factors. The integrity of RNA contained in the original sample, storage conditions, the efficacy of various reagents and enzymes used in RNA extraction, purification, reverse transcription and the actual qPCR, and the type of thermal cycler used are examples of factors that affect the accuracy of qPCR data. In order to take these sources of variation into account, an internal control (reference gene) is commonly used [1].

Reference genes need to have uniform transcript abundance across the different groups being compared (e.g, across treatments, developmental stage, conditions, etc). As such, normalizing transcripts abundance of target genes over that of a reference gene should, in theory, eliminate artifactual variation. It is clear however, that to date, not a single universal reference gene has been found, if it even exists at all [2]. Very often, genes that are stably expressed between treatments in one study system are found to be regulated in another. As such, careful consideration must be given to selecting and validating candidate reference genes [1].

More recently, many studies have employed a combination of multiple reference genes as normalizers [3-5]. This method commonly utilizes software packages, e.g., *GeNorm *[6], *BestKeeper *[7] and *NormFinder *[2], that are specifically designed to determine the most stable normalizer (i.e., with the least transcript abundance variability) amongst a set of candidates, and/or the most stable combination of genes based on their geometric averages. It is argued that the variation in the geometric average of several acceptably stable genes is usually smaller than that of any one of those genes alone, thus increasing normalizer stability [2]. Unfortunately, most of these studies only mention which genes are considered best by the software without validating if the genes are 'good enough'.

At the heart of the issue for most researchers, is whether or not reference genes are differentially regulated between compared groups (e.g., treatment, developmental stage, etc) [2]. In this study, we will illustrate the importance of validating the outcomes of these three reference gene selection programs. We do this by testing the transcript levels or normalization factor values (*NF *calculated in *GeNorm*) of software-selected reference genes for differences between sample groups using traditional non-parametric statistics (Mann-Whitney) and REST 2009 (Corbett Research Pty. Ltd. and Pfaffl, 2009). REST 2009 is a software application specifically created to statistically test qPCR data for pairwise differences between groups. For our purpose, we use the New Zealand shortfinned eel (*Anguilla australis*) as our study system.

Freshwater eels (*Anguilla *spp.) are catadromous teleosts that spawn in the deep-ocean [8-10]. The current study will evaluate the suitability of six candidate reference genes (*eef1*, *actb*, *odc1*, *18s*, *l36*, *nop14*; Table 1) for qPCR analysis in the New Zealand shortfinned eel. We examined the stability of mRNA abundance of candidate reference genes in two tissue types (liver and ovary) between three groups of fish: juvenile sedentary (yellow) eels, migrating adult eels (silver) and 11-ketotestosterone (11-KT) implanted yellow eels (11-KT group). We chose to compare these three groups due to our research interests regarding the transformation of yellow to silver eels (silvering). Silvering involves systemic changes to the physiology, morphology and behavior of eels, a necessary step before final sexual maturation and migration [11-13]. It is now well known that exogenous 11-KT implantation of yellow eels induces morphological and physiological changes consistent with silvering, in particular liver and ovary enlargement, and sexual development [14,15]. Three candidate genes were selected due to their common usage as references in many study systems (*eef1*, *actb*, *18s*) and the remaining three because of our prediction that their mRNA levels should be stable due to their 'housekeeping' roles (Table 1; *odc1*, *l36*, *nop14*). Using three software applications (*GeNorm, BestKeeper *and *NormFinder*), we will determine the best reference gene candidate based on overall transcript stability and across the three groups. We will also validate these selected genes statistically and evaluate for differences between seasons. To our knowledge, this study is the first to formally select suitable reference genes for an anguillid fish and to subject the suitability of software-selected genes to scrutiny in any teleost.

**Table 1:** Candidate reference genes and their functions

Gene name	Gene abbreviations	Function
Elongation factor-1α	*eef1*	Catalysation of GTP-dependent binding of amynoacyl-total RNA to the ribosome; translational factor
Β-actin	*Actb*	Component of the cytoskeleton and mediates cell motility
Ornithine decarboxylase 1	*odc1*	Involved in the urea cycle as rate limiting enzyme in polyamine synthesis, carboxylating L-onithine into diamine putrescine
18 s ribosomal RNA	*18s*	Part of the small ribosomal subunit
60 S ribosomal protein L36	*l36*	Smallest protein in the large subunit of the ribosome
Nucleolar Protein 14	*nop14*	Processing pre *18 s *rRNA and export of 40 s pre-ribosomal unit to cytoplasm

## Results

### qPCR assay validations

All qPCR assays except that for *eef1 *of 2008 ovary samples (efficiency of 94%) had amplification efficiencies between 95% and 105%, and R^2 ^values of ≥ 0.985. All qPCR assays produced a single amplicon as shown by single peaks during melting curve analyses. The identities of qPCR products were further confirmed through sequencing.

### Analyses of transcription stability

**Figure 1:**
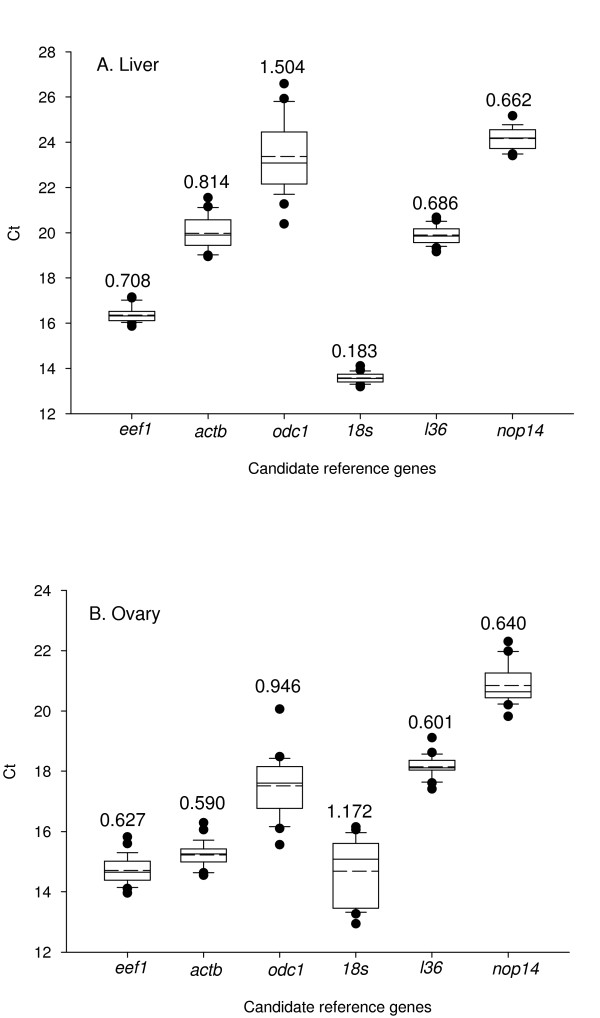
**Overall variability of mRNA levels of candidate reference genes in liver (A) and ovary (B)**. Data are based on the threshold cycle (Ct) values of qPCR of reference gene candidates on liver and ovary samples taken in summer 2009. Box-and-whisker plots denote median, upper and lower quartiles, and 10^th ^and 90^th ^percentiles of data. Dashed lines within bars denote the means. Values above bars denote *M-value *as calculated by *GeNorm *as a measure of overall stability. Lower values denote lower transcript abundance variability, and thus suitability as a reference gene.

**Figure 2:**
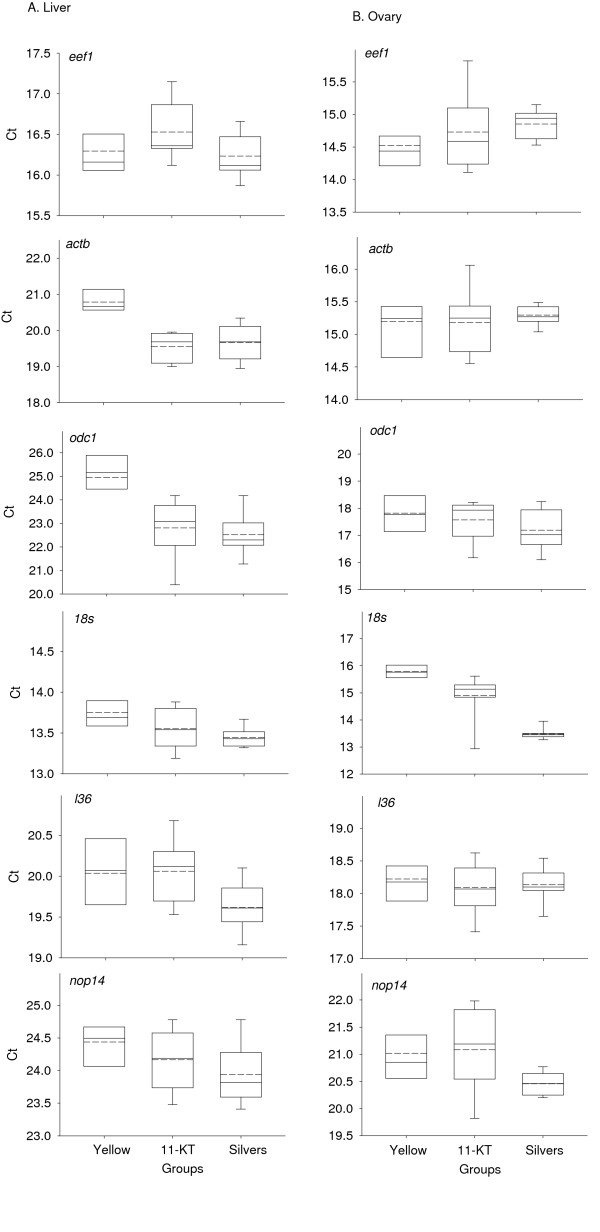
**Intergroup transcript abundance variability of each candidate reference genes in liver (A) and ovary (B)**. Data are based on the threshold cycle (Ct) values of qPCR of reference gene candidates on liver and ovary samples taken in summer 2009. Box-and-whisker plots denote median, upper and lower quartiles, and 10^th ^and 90^th ^percentiles of data. Dashed lines within bars denote the means. A suitable reference gene should have Ct values that are similar across the three groups (e.g., *l36 *in ovary).

Examination of threshold cycle (Ct) values showed considerable variability among the different candidate reference genes (Figure 1), with different patterns between liver and ovary. The most stable and variable genes in the liver were *18 s *and *odc1*, respectively, and in the ovary, *actb *and *18s*, respectively. Based on the *M *value calculated in *GeNorm*, mRNA levels of all candidate genes except *18 s *were more stable in the ovary than liver, a pattern that is replicated when calculated by *NormFinder *based on *Stability value *(data not shown). In general, the rankings obtained by *GeNorm *and *BestKeeper *correspond well with those based on this cursory analyses of overall mRNA levels (see below). Certain genes showed strong indications of being regulated, such as *actb *and *odc1 *in the liver and *18 s *in the ovary (Figure 2).

For liver samples, there is a broad agreement among the three programs, particularly between *GeNorm *and *BestKeeper *(Table 2). Thus, *18 s *was consistently the top-ranked or part of the top-ranked pair of genes while *odc1 *was consistently the worst or second-worst performing gene for liver and ovary. *GeNorm *and *BestKeeper *gave identical decreasing ranking order for *l36*, *nop14*, *actb *and *odc1*. *GeNorm *and *BestKeeper *also selected *eef1 *as the second best and part of the best pair of genes, but *eef1 *was considered the second worst by *NormFinder*.

**Table 2:** Ranking of reference gene candidates based on overall transcription stability.

	Liver	Ovary
GeNorm*[*M *value] (without *18s*)	*eef1*-*18 s *[0.316] <*l36 *<*nop14 *<*actb *<*odc1* (*eef1*-*l36 *[0.330] <*nop14 *<*actb *<*odc1*)	*eef1*-*actb *[0.188] <*l36 *<*nop14 *<*odc1 *<*18s*
		
*BestKeeper *[SD *x-fold*]	*18 s *[1.13] <*eef1 *[1.19] <*l36*<*nop14*<*actb *<*odc1*	*l36 *[1.28] <*nop14 *<*actb *<*eef1 *<*18s *<*odc1*
		
*Normfinder *[Stability value] (best pair)	*18 s *[0.183] <*nop14 *[0.189] *actb *<*l36 *<*eef1 *<*odc1* (*actb*-*l36 *[0.100])	*nop14 *[0.125] <*actb *<*l36*<*eef1 *<*18s *<*odc1* (*nop14*-*actb *[0.098])

*GeNorm does not select the single best gene but the best pair of genes.

Rankings were based on overall transcription stability parameters as calculated by *GeNorm *(*M-value*), *BestKeeper*, (SD *x-fold*) or that calculated by a model based approach by *Normfinder *(*Stability value*). High transcript abundance of *18 s *may make it unsuitable as a reference gene, therefore where *18 s *had been selected by *GeNorm *as part of the best pair of genes, an additional analysis was conducted without *18 s *(results in parantheses). Values in boxed parentheses [ ] indicate indices of stability of the top ranked gene or pair of genes, or the second highest if the top ranked gene was *18s*. Lower scores denote greater transcription stability and suitability as a reference gene. Both *GeNorm *and *Normfinder *select the best pair combination of reference genes.

For ovary samples, there is only a moderate agreement between the three programs (Table 2). *actb *was ranked in the top half by all programs, and *nop14 *in the top two selected by *BestKeeper *and *NormFinder*. In the ovary samples, *18 s *is consistent in being the worst or second-worst performing gene

### Validation of reference genes

Statistical analyses based on REST 2009 and Mann-Whitney provided virtually identical conclusions, with the only exception being those for *18 s *liver on 2008 data (Table 3). Indeed, Mann-Whitney and REST 2009 showed a significant and not-significant difference between yellow and silver groups, respectively.

**Table 3:** Pair-wise statistical analyses of the inter-group stability of selected reference genes.

	Group comparisons	Yellow vs 11-KT		Yellow vs Silver		11-KT vs silver	
**Tissue**	**Reference genes**	***p *based on REST 2009**	***p *based on Mann-Whitney**	***p *based on REST 2009**	***p *based on Mann-Whitney**	***p *based on REST 2009**	***p *based on Mann-Whitney**

2009							
Liver	***eef1*-*18 s *(*GeNorm*)**	**0.898**	**0.815**	**0.071**	**0.074**	**0.067**	**0.190**
	*eef1*-*l36 *(*GeNorm*)	0.457	0.481	0.115	0.167	0.012	0.031
	*18 s *(*BestKeeper*, *Normfinder*)	0.085	0.093	< 0.001	0.001	0.243	0.297
	***eef1 *(*BestKeeper*)**	**0.187**	**0.114**	**0.651**	**1.000**	**0.060**	**0.094**
	*nop14 *(*NormFinder*)	0.224	0.321	0.040	0.059	0.105	0.387
	*actb*-*l36 *(*Normfinder*)	0.002	0.002	< 0.001	< 0.001	0.238	0.297

Ovary	*eef1*-*actb *(*GeNorm*)	0.704	0.073	0.281	0.093	0.525	0.546
	***l36 *(*BestKeeper*)**	**0.533**	**0.673**	**0.638**	**0.673**	**0.784**	**0.863**
	*nop14 *(*NormFinder*)	0.850	0.963	0.007	0.011	0.030	0.050
	*nop14*-*l36 *(*NormFinder*)	0.899	0.673	0.079	0.139	0.135	0.136

2008							
Liver	***eef1*-*18 s *(2009 best)**	**0.082**	**0.079**	**0.892**	**1.000**	**0.166**	**0.113**
	*eef1 *(2009 2^nd ^best)	0.002	0.004	0.283	0.356	0.011	0.019
	*eef1*-*l36 *(*GeNorm*)	0.006	0.006	0.869	0.905	0.006	0.006
	*18 s *(*BestKeeper*)*	**0.308**	**0.549**	**0.422**	**0.053**	**0.884**	**0.063**
	*nop14 *(*Normfinder*)	0.047	0.053	0.038	0.035	0.001	0.003
	*nop14*-*actb *(*Normfinder*)	0.343	0.549	0.002	0.003	0.027	0.024

Ovary	***l36 *(2009 best)**	**0.307**	**0.321**	**0.924**	**0.730**	**0.321**	**0.298**

* *18 s *can only be considered the best for 2008 liver with qualifications as Mann-Whitney test showed significant difference between Yellow and Silver groups.

The top ranked candidate reference genes as selected by *GeNorm*, *BestKeeper *and *NormFinder *were validated using REST 2009 (QIAGEN 2009; see text for explanation) and Mann-Whitney. To reduce Type II error, differences are considered significant at *p *≤ 0.055 (see text for further explanation). Significant difference between any two groups indicates unsuitability as reference genes due to lack of stability across groups. Figures in bold denote the best reference gene(s) based on inter-group stability. The second best option is also denoted in bold when the best included *18 s *(e.g, liver). The best or second best reference gene(s) were applied to the 2008 data. Where it performs poorly (e.g., *eef1 *liver data), the programs were used to select the top ranked candidate gene based on 2008 data, with the statistical validation of the results also shown above.

Most of the genes or pairs of genes selected by the three programs were not sufficiently stable among liver samples, showing strong regulation with treatment (yellow vs 11-KT) or developmental stage (yellow vs silvers) (Table 3). Only the *eef1*-*18 s *combination and *eef1 *by itself were sufficiently stable across the three groups, and no significant pairwise differences were detected by REST 2009 or Mann-Whitney (Table 3). As indicated by the higher *p *values in two pairwise comparisons, the *eef1*-*18 s *combination is more stable than *eef1 *by itself. However, the high transcript abundance of *18 s *may render it unsuitable as reference for lowly expressed genes. In these cases, *eef1 *would be a suitable reference.

For ovary, all of the genes or pairs of genes selected by the programs were sufficiently stable to act as references, except for *nop14 *which seems to be down-regulated in silver eels (Table 3; Figure 2). Among the selected reference genes, a single gene - *l36 *- was shown to perform best in terms of stability by yielding the lowest probability of a statistical difference between two pairwise comparisons.

The best reference genes identified from 2009 data were applicable for 2008 data for both liver and ovary (Table 3). The 2008 *eef1*-*18 s *combination for liver and *l36 *for ovary were suitable for use as reference genes. However, we found significant differences in two sets of pairwise comparisons for liver *eef1*, making it unsuitable as a reference gene on this data set. To find out if any other reference genes are suitable, we ran the reference gene selection programs on the 2008 liver data and validated the selection statistically. The results indicated that *18 s *used as a single reference gene was the only other viable option for 2008 liver samples, as validated by REST 2009. However, it should be noted that Mann-Whitney analyses showed a significant difference in *18 s *mRNA levels between yellow and silver groups.

## Discussion

In this study, we have for the first time selected reference genes that are suitable for use in qPCR of ovarian and liver samples from an anguillid eel. Our results show that for studies on the silvering of freshwater eels and the involvement of 11-KT in this event, the transcript levels for *l36 *and the combination of *eef1 *and *18 s *are suitable for use as references in ovary and liver tissues, respectively. More importantly, we have for the first time demonstrated a method to validate the conclusions made by reference gene selection programs. Indeed, our validation method has surprisingly shown that a single gene (*l36 *in ovary) can perform better in terms of intergroup stability than a combination of genes.

Due to the widespread use of qPCR, it is unsurprising that reference gene selection programs are commonly used and form a critical element of publication of qPCR data. However, this study has highlighted several reasons for why external validations of the conclusions drawn from such programs are needed.

First, just as there is no single universal reference gene, no single program could provide the optimal reference genes for all situations. As can be expected from the different algorithms and assumptions employed, the three programs often do not agree on the best gene or rankings of genes [16]. The optimal gene(s) for liver and ovary, and even liver itself between seasons, were selected by different programs. We highly recommend that researchers interested in computer-assisted reference gene selection use multiple programs. It is encouraging that this approach has been adopted by many similar studies [16-20].

Second, the optimal reference genes selected by a program can only be as good as their candidates. In our case, ovary had greater overall transcript stabilities than liver for all but one gene (*18s*). This is consistent with our findings that most program-selected reference genes performed satisfactorily for ovary, while the opposite was found for liver where only two out of six selected genes passed statistical validation.

Third, differences in study design or context may affect the performance of reference genes, even within the same species. In this study, we show that year and season affected the performance of *eef1 *as a reference gene. It performed satisfactorily for samples taken in summer 2009 but showed a strong effect of treatment in the autumn 2008. Changes in reference gene suitability due to differences in study design (e.g., *in vivo *vs *in vitro*) or developmental stage have been reported in many studies (e.g., locusts [17], cerebral ischaemia in rats [21], channel catfish [22]). Thus researchers need to be vigilant in validating the reference genes used in every study.

All three potential problems described above could be mitigated by using an independent statistical validation method. REST 2009 was written by the same group that produced *BestKeeper*, and its conclusions very closely matched the independent Mann-Whitney tests which strongly suggest that it is a suitable validating program. We therefore, recommend the use of REST 2009 because its specific design for qPCR data analysis circumvents the difficulties of parametric statistical analyses on data based on proportions, such as those generated by qPCR [23]. However, other methods of qPCR data analyses are available (reviewed by Pfaffl et al 2009) and may be equally or more suitable, such as when analyses other than pair-wise comparisons are required.

There has been a realization that a great number of publications of qPCR data do not include sufficient information to allow for evaluation of reliability of the results [22,24]. One of the key description pertains to the selection of reference genes (selection procedure, validity, etc), yet, this information is often not provided. Fewer still indicate whether or not reference genes are stably transcribed across the compared groups, with many simply citing previous research that used the same genes in a similar study system. Our findings convincingly show that such approaches are inadequate as a matter of due diligence.

## Conclusions

We suggest that all reporting of normalized qPCR data include the following information: 1) Justification/rationale for the selection of the reference genes used (e.g., selected using computer programs, previous use in a similar study system, etc), and 2) whether or not the mRNA levels of reference genes are different between the experimental groups.

## Methods

### Sampling

The main sampling was conducted in the austral summer of 2009 (February - March 2009). Female yellow eels (n = 20) and silver eels (n = 10), weighing between 800-1200 g, were caught by fyke net from Lake Ellesmere, Canterbury, New Zealand. Within 48 h of capture, they were intraperitoneally implanted with passive-integrated-transponder tags. At the same time, the silver and half of the yellow eels were given placebo implants (30 mg, 95% cholesterol, 5% cellulose), while the remaining yellow eels received 11-KT implants (same size and composition as placebo, but with 1 mg of 11-KT). Eels were then transferred to a salmon raceway (35 m × 5 m; New Zealand King Salmon Hatchery, Tentburn, Canterbury, New Zealand) for 28 days to allow the effects of 11-KT to be manifested in treated eels. Water was kept at a depth of c.30 cm with a unidirectional gentle flow. At the end of the exerperimental period, all eels were sacrificed with an overdose of anaesthetic (benzocaine) and liver and ovary samples taken and flash frozen in liquid nitrogen until storage in a -70°C freezer.

Our 2009 study was a refinement of one conducted in 2008. This earlier study was carried out later in autumn (April - May 2008), with a shorter captivity period (21 days). Data from the 2008 study were used to evaluate if selected reference genes (on the basis of 2009 data) were suitable for use between seasons.

### RNA extraction and cDNA synthesis

Total RNA was extracted from frozen tissue samples (< 100 mg) using TRIZOL (Invitrogen) according to the manufacturer's instructions, and quantified using a spectrophotometer (NanoDrop ND 1000, Thermo Fisher Scientific). The quality of RNA samples were ascertained using an Agilent Bioanalyzer 2100 (Agilent Techonologies), according to the manufacturer's instructions. All RNA samples were shown to be of high integrity, with clear and distinct peaks at the 18 S and 28 S areas. Liver RNA samples all had RNA integrity numbers (RIN) of ≥ 9.0. However, we detected very high amounts of small RNA in ovary samples, particularly for those of yellow eels (see Additional file 1: representative electropherograms of ovary and liver RNA). This observation was consistent and was also encountered by a colleague (Dr Yuichi Ozaki) in his ovary samples. As a result, the software could not calculate RIN for ovary samples of yellow eels, but could do so for those of silver eels (RIN ≥ 8.0). Five micrograms of total RNA were then treated with Turbo DNA-free (Ambion) according to manufacturer's instructions to minimize potential genomic DNA contamination, and quantified using spectrophotometry. Information from the manufacturer stated that DNAse treatment were able to reduce DNA contamination in RNA samples by 5.4 million fold (Ambion 2009). Our own analyses conducted by comparing the Ct of DNAse treated and untreated liver cDNA showed a 512 fold reduction (99.8% reduction). One microgram of DNAse treated total RNA was then reverse-transcribed using High Capacity cDNA Reverse Transcription Kit with RNAse Inhibitor (Invitrogen) using random hexamer primers. The resulting 20 μl cDNA volume was finally diluted with 60 μl deionized water to make a cDNA concentration of 12.5 ng original total RNA/μl.

### Cloning of target genes for sequencing and making of standards

We have cDNA sequence information for elongation factor-1 (*eef1*) and β-actin (*actb*) for *A. australis *from previous work (e.g., Lokman et al 2007). Primers for *18 s *rRNA were designed based on the published sequence for *A. australis *(FM946133). Expressed sequence tags obtained after suppressive subtractive hybridization of two ovarian libraries from *A. australis *(Lokman, unpubl. data) yielded the sequence information for eel orthologues of *l36*, a ribosomal protein, and nucleolar protein-14 (*nop14*). Degenerate primers for ornithine decarboxylase-1 (*odc1*) were designed on the basis of conserved motifs among *odc1 *orthologues from several teleost fish. Complementary DNAs were cloned into plasmids for sequencing or development of qPCR standards. Cloning parameters (primer sequences, melting temperature, amplicon sizes and identity at the nucleotide levels with known sequences) are shown in Table 4.

**Table 4:** Cloning parameters for candidate reference genes*

Gene	Primers (5 μM)	Accession number	Anneal. temp. (°C)	Frag. size (bp)	Seq. identity (ref. species, accession number)
*eef1*^‡^	FW: atgggaaaggaaaagatccacatca RV: tcaagcttcttgccagaacgacggt	HM367094	52	1163	99% (*A. anguilla*, EU407825.1)
*actb*	FW: agagctacgagctgcctgac RV: cgggtggggcaataatct	HM357464	60	288	92% (*A. Anguilla*, DQ493907.1)
*odc1*	FW: caratgatgacnttygayws RV: ccrtcrttnacrtartacat	HM357466	60	581	84% (*Salmo salar*, BT044794.1)
*18s*	FW: gtacacacggccggtacagt RV: ggtaggcgcagaaagtacca	FM946133	60	302	100% (*A. Australis*, FM946133)

*l36 (HM357467) and *nop14 *(HM357468) sequences were obtained whilst sequencing expressed sequence tags from a suppressive subtractive hybridization library (Lokman, unpubl. data).

^‡^From Lokman et al (2007).

Partial target gene cDNAs were amplified from ovary or liver templates using PCR (Bioline: Biotaq Red DNA Polymerase, 10× NH_4 _Buffer, 50 mM MgCl_2 _Solution). PCR products were electrophoresed, and amplicons of expected sizes extracted from the gel using the MinElute Gel Extraction Kit (Qiagen). Complementary DNAs were then ligated into pGEM T-Easy Vector (Promega) according to manufacturer's instructions. Ligated plasmids were transfected into *Escherichia coli *XL-1 Blue, grown overnight and single colonies amplified in 2YT medium. Plasmid was isolated using the Qiaprep Spin Miniprep Kit (Qiagen) and sequenced (Allan Wilson Centre Genome Service, Palmerston North, New Zealand) using M13 forward and reverse primers. Sequence identity was confirmed using the Basic Local Alignment Search Tool in the NCBI database http://blast.ncbi.nlm.nih.gov/Blast.cgi. Sequences were aligned with those known for the species or those of other teleosts. All cloned fragments were shown to have high identity with known sequences (Table 4).

Purified plasmids were linearized using restriction enzyme (*Spe I *or *Nco I*, Roche Diagnostics), and subsequently mixed with 10 volumes TE buffer and 10 volumes phenol:chloroform:isoamyl-alcohol (PCI; 25:24:1) to inactivate enzymes and remove protein residues. Following centrifugation and precipitation by sodium acetate and ethanol [25], plasmid-insert constructs were dissolved in TE buffer, quantified by spectrophotometry, and serially diluted for use as standards in qPCR (10^0 ^- 10^-6 ^ng linearised plasmid/μl).

### qPCR

Primers for qPCR (Table 5) were nested within the cloned cDNA of each target gene. Due to lack of genomic information, we were not able to design primers spanning intron-exon boundaries. However, as RNA was DNAse-treated prior to reverse transcription, genomic contamination is kept at negligible levels. All samples for a particular tissue type were assayed in duplicate on a single 96-well plate along with standards and a no-template control (deionized water), thus eliminating inter-plate variability. Due to the high concentration of *18 s *RNA in each sample, qPCR for this target was conducted on cDNA samples diluted 100× in deionized water. Assays were conducted in 20 μl reaction volumes using Express SYBR GreenER reagent (Invitrogen) on a MX3000P (Stratagene, LaJolla, CA, USA) thermal cycler with a thermal profile of 50°C for 2 minutes, 95°C for 2 minutes, followed by 40 cycles of denaturation at 95°C (30 s), annealing between 62-64°C (30 s; see Table 5 for specific annealing temperatures) and extension at 72°C (30 s). At the end of 40 cycles, a final denaturation step at 95 °C (1 min) was followed by a melting curve analysis. The melting curve analysis according to the manufacturer's information involves a 1-minute melting segment of 95°C, followed by a 30 second minute annealing step of 55°C. The temperature is then increased in a stepwise fashion at 1°C increments until 95°C, with the temperature held at each step for 30 seconds. Fluorescence data is collected at the end of each 30 second step. The data is presented as the first derivative of the fluorescence level (-R'(T)), with each peak in the data representing a qPCR product. Assays for each reference gene candidate produced a single peak.

**Table 5:** qPCR parameters for candidate reference genes

Gene	Forward primer (conc.)	Reverse primer (conc.)	Annealling Temp. (°C)	Amplicon Size (bp)	Amplification efficiency			
					Liver		Ovary	
					2008	2009	2008	2009
*eef1*	cccctgcaggatgtctacaa (200 nM)	agggactcatggtgcatttc (200 nM)	64	152	98%	95%	94%	95%
*Actb*	aatcctgcggtatccatgag (250 nM)	gccagggatgtgatctcttt (250 nM)	62	154	101%	105%	104%	102%
*odc1*	ggacgactcaaaggcagtgt (500 nM)	ccaatgtccagaagggtcat (500 nM)	64	234	96%	104%	96%	104%
*18s*	ggatgcgtgcatttatcaga (200 nM)	cgaaagttgatagggcagaca (200 nM)	64	145	103%	105%	101%	101%
*l36*	cctgaccaagcagaccaagt (250 nM)	tctctttgcacggatgtgag (250 nM)	62	160	101%	102%	102%	101%
*nop14*	gagagcgagagaggctgaag (250 nM)	tttccactctccctcctgtg (250 nM)	62	185	101%	101%	100%	100%

We confirmed the identities of the qPCR products by the following method. We first collected representative ovary samples at the end of a qPCR assay for each reference gene candidate, which were then gel-electrophoresed. Consistent with the results of the melting curve analyses, only single bands at the expected sizes were visible for each reference gene candidate (data not shown). DNA fragments were then extracted from the gel using the MinElute Gel Extraction Kit (Qiagen) and sequenced at the University of Otago Department of Anatomy and Structural Biology.

### Selection of best reference genes

We used three software applications specifically designed to measure transcript stability and to identify the best candidate gene or best gene combination from a selection of candidate genes, *GeNorm*, *Bestkeeper *and *NormFinder*. All three programs were written as Microsoft Excel Visual Basic macros and are freely available from the internet.

*GeNorm *is by far the most widely used program among the three with 1128 citations, compared to 126 for *BestKeeper *and 92 for *NormFinder *as of July 2008 (Vandesompele 2009). *GeNorm *considers the best reference genes as those which show the most stable transcript levels (relative quantities based on standards or delta-Ct) across all samples, disregarding *a priori *comparative groupings. Stability is measured as the average pairwise variation (SD of log-transformed ratios) of a candidate gene with each of the other candidate genes (*M *value). Lower *M *value denotes higher stability. The program calculates the candidate gene with the highest transcript stability by a stepwise elimination of the least stable gene (highest *M *value) until only two candidate genes are left. *GeNorm *then calculates the geometric means of this pair of genes to become the normalization factors (NF) that can be used to normalize the transcript levels of target genes. *GeNorm *also calculates the optimal number of reference genes, which is useful when geometric means of the two best reference genes are not sufficiently stable, or when the addition of another gene increases stability.

Unlike *GeNorm*, *BestKeeper *uses raw Ct as inputs, rather than relative quantities. The program has as its assumption that genes that are stably transcribed, and thus suitable to act as references, should be highly correlated with each other. Therefore, in addition to ranking suitable reference gene candidate based on SD of Ct values, it also performs repeated pairwise correlation analyses of all candidate genes. The geometric means of Cts of genes that are highly and significantly correlated with each other are then calculated as an index (the equivalent of NF in *GeNorm*).

*NormFinder *uses a different approach to selecting the best reference genes. Instead of measuring overall transcript stability, it takes into account possible variation across the different sample groups of interest. It uses a "model based approach to estimation of expression variation" (Andersen et al 2004) to select suitable reference genes. In this algorithm, intra- and inter-group variations are estimated, combined into a stability value and the candidate genes ranked accordingly. *NormFinder *also calculates the optimal combination of two candidate reference genes for use as normalizers particularly with regard to intergroup stability. In this case, we used *GeNorm *to calculate NF for the gene combination.

### Statistical validation

All data were analyzed according to the instructions for each program. The best reference gene or combinations of genes that were selected by the programs were then statistically analyzed for differences between treatments or stages. The three groups being compared (yellow, silver and 11-KT) do not represent *levels *within a *factor*. The relationship between the three groups are that of *stage *(yellow vs silver), *treatment *(yellow vs 11-KT) and *natural or artificial silvering *(silver vs 11-KT). Therefore, it is not appropriate to use ANOVA or its equivalents.

Differences between groups were detected using non-parametric pairwise comparisons. We used REST 2009 (Corbett Research Pty. Ltd. and Pfaffl, 2009), a program specifically designed to conduct pairwise comparisons by using randomization and bootstrapping techniques. It allows the user to input multiple target genes, as well as reference genes. Based on its calculated normalized values of the target gene(s), the program then produces an output that tells the user the direction of difference between the groups, as well as the p value. The program is able to normalize the data over combinations of reference genes (based on the geometric averages). Therefore, REST 2009 is not a program originally designed to measure reference gene stability. However, it can provide a statistical output for stability between treatment groups for single genes input as 'reference', but not for combinations of reference genes.

Therefore, we had to 'trick' REST to measure the stability of reference gene combinations by including a dummy target gene variable which was given a uniform Ct value of 15. The candidate reference genes (e.g., *actb*, *eef1*, etc) were input as 'reference', the dummy gene was input as 'target'. Thus, the dummy gene was not meant to represent another normalizer, but merely a mathematical device to allow the stability of the combination of reference genes to be assessed. This method assumes that a stable combination of reference genes would not show a stably (uniformly) transcribed target gene - in this case the dummy gene - as being regulated. By testing for differences between groups for the dummy variable, we thus calculated the stability of the reference genes. In order to treat our analyses consistently, we utilized the dummy gene even when only one reference gene was being assessed (e.g., *l36 *for ovary). The results were virtually identical to when we did not use the dummy gene device (data not shown).

Due to this unorthodox method, we also utilized Mann-Whitney test in SPSS 17 to directly test for differences in transcript abundance or NF. Due to the multiple pair-wise comparisons, the Bonferroni correction dictates that statistical significance should be set at p < 0.016. However, since the onus is on minimizing Type II error, we have set our significance at a conservative level of p < 0.055. When *18 s *was selected as the best gene by *BestKeeper *or *NormFinder*, the second best gene was also statistically validated, because *18 s *may not be a suitable reference for many target genes.

### Validation of results using 2008 data

Genes, or combination of genes that have been selected by the software applications as best were used as references in the 2008 data. We also tested their stabilities by statistically examining the differences in mRNA levels or NF between groups.

### Ethical approval

All experiments were undertaken in compliance with the University of Otago Animal Ethics Committee and New Zealand national standards for animal research.

## Authors' contributions

ANS conducted the sampling, cloning of candidate genes, RIA, qPCR, data analyses and manuscript preparation. PML conducted the SSH and provided plasmids for two candidate genes, provided sequences for *eef1 *and *actb *through previous research, and assisted in the manuscript preparation. Both authors have read and approved the final manuscript.

## Supplementary Material

Additional file 1 **Representative electropherograms of ovary and liver RNA**. This additional file contains examples of electropherograms of ovary and liver RNA in order to highlight the high concentrations of small RNA in the ovary samples, which prevented the calculation of RNA integrity numbers (RIN) for ovary RNA samples of yellow eels. Click here for file
