# Supplementary material for: The use of reference gene selection programs to study the silvering transformation in a freshwater eel Anguilla australis: a cautionary tale
Source: BMC Mol Biol. 2010 Sep 22;11:75. doi: 10.1186/1471-2199-11-75 (PMC2949605; doi:10.1186/1471-2199-11-75)
Supplement: Additional file 1 — Representative electropherograms of ovary and liver RNA. This additional file contains examples of electropherograms of ovary and liver RNA in order to highlight the high concentrations of small RNA in the ovary samples, which prevented the calculation of RNA integrity numbers (RIN) for ovary RNA samples of yellow eels. [file 1471-2199-11-75-S1.PDF]

Additional file 1: representative electropherograms of ovary and liver RNA

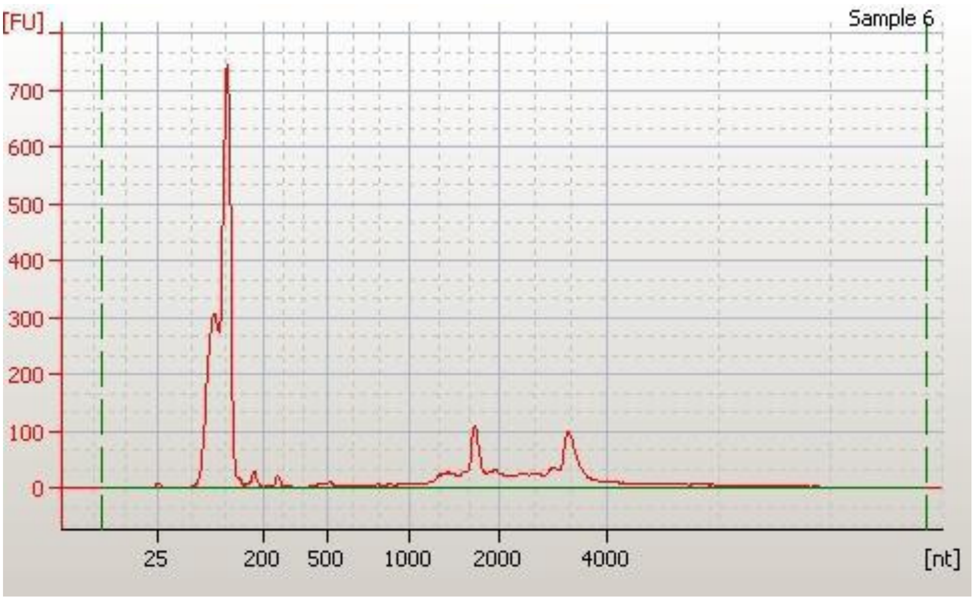

Additional figure 1: Representative electropherogram output from Agilent Bioanalyzer 2100 for RNA of yellow eel ovary. Note that the very high concentration of small sized RNA (between 25 and 200 nt) relative to that of the 18S (between 1000 and 2000 nt) and 28S (between 2000 and 4000 nt) rRNA, which prevents the software from calculating the RNA integrity number (RIN). Also note the distinct peaks in the 18S and 28S regions, indicating high RNA integrity in the sample.

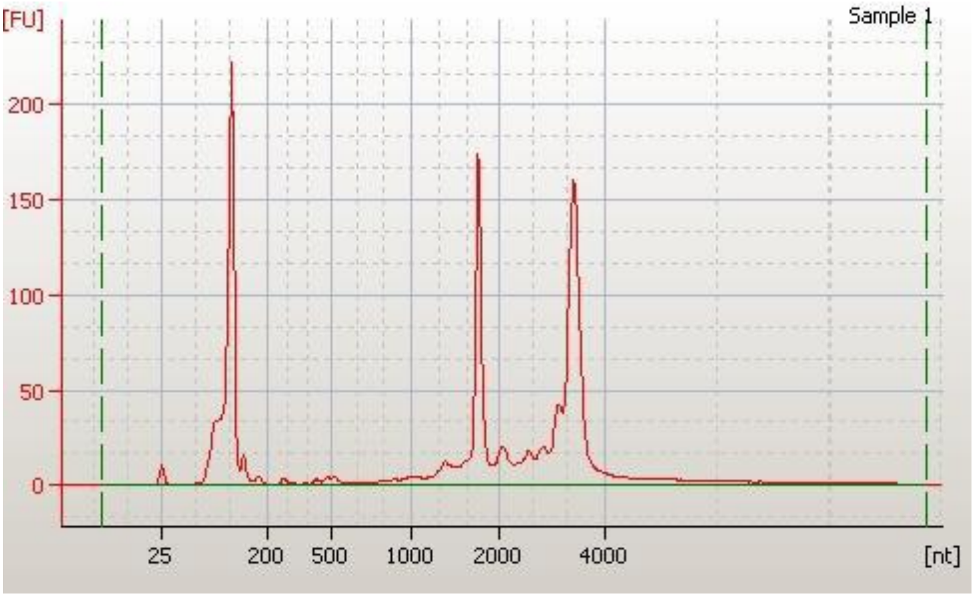

Additional figure 2: Representative electropherogram output from Agilent Bioanalyzer 2100 for RNA of silver eel ovary. Note that the concentration of small sized RNA (between 25 and 200 nt) relative to that of the 18S (between 1000 and 2000 nt) and 28S (between 2000 and 4000 nt) rRNA is not so high as in samples from yellow eel ovary, thus still allowing the software to calculate the RNA integrity number (RIN). For silver eel ovary samples, the RINs were greater than 8.0.

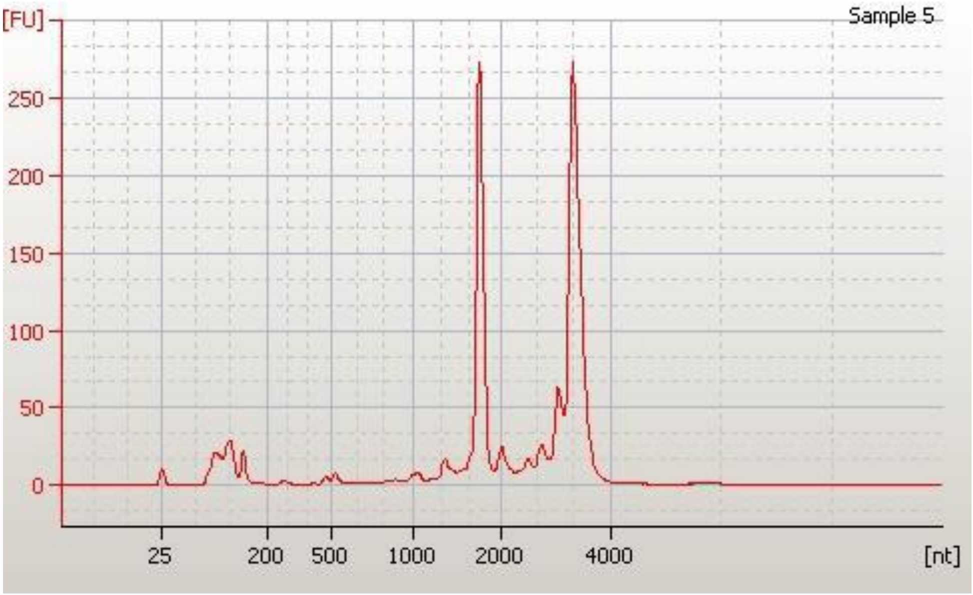

Additional figure 3: Representative electropherogram output from Agilent Bioanalyzer 2100 for RNA of silver eel liver. Note that in liver samples and the brain (Yuichi Ozaki, unpubl. Data), the amounts of small sized RNA (between 25 and 200 nt) relative to that of the 18S (between 1000 and 2000 nt) and 28S (between 2000 and 4000 nt) rRNA are low, unlike ovary samples. The RNA integrity numbers (RINs) for liver samples were at least 9.0.
